# Ubiquitin Ligases CBL and CBL-B Maintain the Homeostasis and Immune Quiescence of Dendritic Cells

**DOI:** 10.3389/fimmu.2021.757231

**Published:** 2021-09-23

**Authors:** Haijun Tong, Xin Li, Jinping Zhang, Liying Gong, Weili Sun, Virginie Calderon, Xiaochen Zhang, Yue Li, Adeline Gadzinski, Wallace Y. Langdon, Boris Reizis, Yongrui Zou, Hua Gu

**Affiliations:** ^1^ Molecular Immunology Research Unit, Montreal Clinic Research Institute, Montreal, QC, Canada; ^2^ Department of Microbiology and Immunology, University of Montreal, Montreal, QC, Canada; ^3^ Department of Biochemistry and Molecular Medicine, University of Montreal, Montreal, QC, Canada; ^4^ Institute of Biology and Medical Science, SooChow University, Jiangsu, China; ^5^ Division of Experimental Medicine, McGill University, Montreal, QC, Canada; ^6^ School of Biomedical Sciences, University of Western Australia, Crawley, WA, Australia; ^7^ Department of Pathology, New York University Langone Medical Center, New York, NY, United States; ^8^ Department of Medicine, New York University Langone Medical Center, New York, NY, United States; ^9^ The Feinstein Institute for Medical Research, Manhasset, New York, NY, United States

**Keywords:** E3 ubiquitin ligase, dendritic cell (DC), FLT3, liver inflammation, homeostasis

## Abstract

Dendritic cells (DCs) are composed of multiple lineages of hematopoietic cells and orchestrate immune responses upon detecting the danger and inflammatory signals associated with pathogen and damaged tissues. Under steady-state, DCs are maintained at limited numbers and the functionally quiescent status. While it is known that a fine balance in the DC homeostasis and activation status is also important to prevent autoimmune diseases and hyperinflammation, mechanisms that control DC development and activation under stead-state remain not fully understood. Here we show that DC-specific ablation of CBL and CBL-B (CBL^-/-^CBL-B^-/-^) leads to spontaneous liver inflammation and fibrosis and early death of the mice. The mutant mice have a marked expansion of classic CD8α^+^/CD103^+^ DCs (cDC1s) in peripheral lymphoid organs and the liver. These DCs exhibit atypical activation phenotypes characterized by an increased production of inflammatory cytokines and chemokines but not the cell surface MHC-II and costimulatory ligands. While the mutant mice also have massive T cell activation, lymphocytes are not required for the disease development. The CBL^-/-^CBL-B^-/-^ mutation enhances FLT3-mTOR signaling, due to defective FLT3 ubiquitination and degradation. Blockade of FLT3-mTOR signaling normalizes the homeostasis of cDC1s and attenuates liver inflammation. Our result thus reveals a critical role of CBLs in the maintenance of DC homeostasis and immune quiescence. This regulation could be relevant to liver inflammatory diseases and fibrosis in humans.

## Introduction

Dendritic cells (DCs) are not only special sentinels that orchestrate immune responses against various pathogens but also important regulators to control immune tolerance and inflammation (1–3). DCs detect pathogens and inflammation cues *via* pattern recognition receptors such as Toll-like (TLRs), NOD-like, and TNF family of receptors (4–7). In the absence of infectious and inflammatory stimuli, DCs are either maintained at the quiescent status or function as regulatory DCs that actively induce immune tolerance (8, 9). However, since the ligands for the pattern recognition receptors, such as commensal microbes and host metabolic or tissue damage products, are constantly present under steady-state, there must be mechanisms that refrain DCs from provoking unwanted immune responses (10, 11). The functional quiescence of DCs is known to depend on intracellular negative regulators for pattern recognition receptor signaling (12, 13). In addition, it has been reported that increased lifespan of DCs or depletion of DCs may exhibit a profound effect on the immune quiescence and the development of autoimmune and myeloid proliferative diseases (14, 15). These findings thus suggest that control of a fine balance in DC homeostasis can be another important dimension to maintain DC functional quiescence under steady-state.

DCs are composed of multiple lineages, including classical or conventional DC (cDC) and plasmacytoid DCs (pDC), and the former are further divided into the subsets of CD8α^+^ cDC (cDC1), CD11b^+^ cDC (cDC2), and tissue CD103^+^ cDC1 (16, 17). CD8α^+^ and CD103^+^ cDC1s are functionally unique because they present antigens not only to CD4^+^ but also to CD8^+^ T cells by a mechanism termed cross presentation (18–20). Lineage specification of cDC1s is controlled by the transcription factor IRF8, which may coordinate with BATF family members (21–23). In addition, Notch signaling has been shown to be necessary for optimizing cDC1 genesis (24). Moreover, while development of both cDC1s and cDC2s requires FLT3 signaling, enhanced activity of PI3 kinase that acts at the downstream of FLT3 may selectively expand cDC1s (25). However, given that FLT3L is constantly present in periphery it is not fully understood how the homeostasis of cDC subsets in the periphery is maintained under steady state.

Chronic liver inflammation is a major cause for liver fibrosis and cirrhosis, a devastating disease that often leads to cancer or death. The pathologic process is usually associated with chronic liver injury as a result of virus infection, toxin stress, alcoholic, nonalcoholic steatohepatitis and fatty liver diseases, and autoimmune diseases (26). While inflammation developed following liver injury is complicated due to the diverse causes, it can be attributed mainly to the activation of liver macrophages, including Kupffer cells and migratory macrophages (27, 28). Activated macrophages may recruit other immune cells to the damage site and activate hepatic stellate cells which then initiate a cascade of extracellular matrix secretion and deposition (26). Although it is well known that DCs are the first line of players to detect the inflammatory alarm and danger signals, the role of DCs in liver inflammation and fibrosis remains largely unclear (29).

CBL and CBL-B (CBLs), two members of the CBL family of E3 ubiquitin ligases, are important in the prevention of autoimmune diseases mediated by T and B cells (30, 31). They regulate T and B cell development, tolerance, and function by modulating the signals delivered by the T and B cell antigen receptors and coreceptors (31, 32). However, in contrast to T and B cells we still know little about the roles of CBLs in DC biology, in particular in the context of inflammation and immune tolerance. In this report, we show that conditional ablation of CBLs, but not CBL nor CBL-B alone, in DCs is detrimental to mice, as the mutant mice manifest severe spontaneous inflammation predominantly in the liver and liver fibrosis. Although most CD4^+^ and CD8^+^ T cells in the mutant mice are activated, lymphocytes are not required for the development of the disease. The mutation significantly alters DC homeostasis, as the mutant mice possess markedly more CD8^+^ cDC1s but not CD11b^+^ cDC2s in peripheral lymphoid organs and CD103^+^ DC1s in the liver. In addition, the mutant DCs exhibit a phenotype of atypical hyperactivation, characterized by high levels of IL-6 and inflammatory chemokines, but normal cell surface costimulatory molecules and MHC-II. Moreover, we show that ablation of CBLs impairs FLT3 ubiquitination, resulting in enhanced FLT3-AKT-mTOR signaling. Blockade of mTOR signaling in the mutant mice rebalances the homeostasis of cDC1s and attenuates the liver inflammation and fibrosis. Thus, our data demonstrate that CBL-mediated protein ubiquitination acts as a critical DC intrinsic regulatory mode to maintain the homeostasis and immune quiescence of peripheral cDCs under steady-state. These results also suggest a potential role of cDCs in human liver inflammation and fibrosis and that modulation of DC homeostasis could be an effective treatment for liver inflammation and fibrosis.

## Methods

### Animals

C57BL/6 mice, B6.SJL mice, RAG1^-/-^ mice were from The Jackson Laboratory. CBL^flox/flox^ and CBL-B^-/-^ mice were described previously (33, 34). CBL^-/-^ CBL-B^-/-^ mice were generated by crossing *Cbl^flox/flox^* and *Cbl-b^-/-^* mice to *CD11c-Cre* transgenic mice (24). RAG1.CBL tko mice were generated by breeding CBL^-/-^ CBL-B^-/-^ mice to RAG1^-/-^ mice. CBL-B^C373A/C373A^ mice were described previously (35). All animal experiments were performed in accordance with the Canadian Council of Animal Care and approved by The Institut de Recherches Cliniques de Montréal (IRCM) Animal Care Committee.

### Antibodies and Flow Cytometry

Total splenic cells, bone marrow cells, or liver infiltrating cells were resuspended in FACS buffer [5% BSA in PBS (PH=7.2)] and stained with the corresponding antibodies on ice for 30 min. Cells were washed twice with FACS buffer and then subjected to analysis on a BD Fortessa or Cyan or to cell sorter purification on a FACS Aria or Moflo. The following antibodies were used for the staining: anti-B220, anti-CD11c, anti-MHCII, anti-CD11b, anti-CD8α, anti-CD4, anti-CD44, anti-CD62L, anti-CD3ε, anti-F4/80, anti-Gr1, anti-CD86, anti-CD80, anti-CD40, and anti-PD-L1 (eBioscience). For BrdU incorporation analysis, mice were injected intravenously with 1 mg of BrdU (BD PharMingen) in DPBS. Cells were then surface stained with corresponding antibodies, and BrdU labeled cells were stained using an anti-BrdU kit according to the manufacturer’s protocols (BD PharMingen).

### Pathological Analysis

Tissues were harvested, fixed in paraffin, cut into 8 µM sections and then stained with Hematoxilin and Eosin (H&E). For liver fibrosis, collagenous components were revealed by Masson’s trichrome staining.

### Bone Marrow DC Culture

FLT3L conditioned medium was collected as the supernatant of FLT3L-secreting B16 melanoma cell culture. BM cells (10^6^ cells/ml) were cultured in different doses of FLT3L conditioned medium at 37°C for 7-9 days, before being harvested for FACS analysis or subjected to cell sorting.

### DC Activation and Cytokine Analysis

Purified DC (10^6^ cells/ml) were cultured in 48-well plate in the presence or absence of 10 µg/ml LPS (Sigma) or 10 µM/ml CpG (Invivogen). After 1-3 days, DCs were harvested and subjected for flow cytometric analysis. Culture supernatants were collected, and cytokines in the supernatants were detected by Enzyme-Linked Immunosorbent Assay (ELISA) using a Ready-Set-Go ELISA kit (eBioscience). In brief, supernatant or serum samples were diluted and incubated in 96-well plates pre-coated with different capture antibodies at 4°C overnight. Plates were washed three times and incubated with horseradish peroxidase (HRP)-conjugated detection antibodies for at 37°C for one hour. After washing with PBS, HRP activity in each well was developed in tetramethylbenzidine (TMB) substrate solution.

### Antigen Presentation and T Cell Proliferation Assay

OVA-specific CD8^+^ (OT-I) or CD4^+^ (OT-II) splenic T cells were purified by EasySepTM Mouse CD8^+^ or CD4^+^ T cell enrichment kit as described in manufacturer’s manual (StemCell Technologies). Purified T cells were labeled with CFSE (ThermoFisher), cocultured with OVA (40 µg/ml) and graded ratios of purified splenic DC subsets in a 96-well plate for 3 days or 4 days. OT-I and OT-II T cells were stained with anti-TCR Vα2, and cell proliferation was determined based on the dilution of CFSE.

### Biochemical Analysis

For FLT3, AKT, and ERK signaling, purified DC subsets were incubated at 37°C without serum and FLT3L for at least one hour. Cells were then stimulated with FLT3L at 37°C for the indicated time, lysed in TNE buffer (50 mM Tris; 140 mM NaCl; 5mM EDTA; 0.5% SDS), and immunoblotting was performed following standard procedures. For immunoprecipitation, proteins were immunoprecipitated by incubating cell lysate with the appropriate antibodies (1-2 µg/1 ml) at 4°C overnight, followed by precipitation of the protein-antibody complexes using protein G agarose (Cell Signaling) for another 1 hour at 4°C. Immunoprecipitates were washed four times with TNE buffer, boiled in 40 µl loading buffer and immunoblotted to a PVDF membrane for western blot analysis. The following antibodies were used for biochemical study: anti-CBL (SantaCruz); anti-CBL-B (Cell Signaling); anti-β Actin (abcam); anti-FLT3 (abcam); anti-pFLT3 (Cell Signaling); anti-pAKT (Cell Signaling); anti-pEKR1/2 (Cell Signaling); anti-HA, anti-IRF8, anti-IRF4, anti-ID2 (Santa Crutz). HRP-conjugated goat anti-rabbit, goat anti-mouse or donkey anti-goat antibody was used as a secondary antibody, respectively. HRP activity was detected using an enhanced chemiluminescence detection system (GE Healthcare).

For FLT3 ubiquitination and degradation assays, 2 µg pcDNA-FLT3 and pcDNA-Ub-HA was co-transfected with 2 µg pcDNA empty vector, pcDNA-CBL or pcDNA-CBL-B to HEK-293 cells by calcium transfection, respectively. Forty-eight hours later, transfected cells were treated with or without FLT3L (100 ng/ml) at 37°C for two hours. For protein degradation assay, new protein synthesis was blocked by Cycloheximide (100 µg/ml) (Cell Signaling). For ubiquitination assay, protein degradation was blocked with chloroquine (40 µg/ml) or MG132 (20 µg/ml) for 15 min before stimulation with FLT3L. FLT3 ubiquitination and expression were then analyzed by Imunoprecipitation and Western blot analysis.

### RNAseq and qPCR

To study the gene expression profiling, CD11b^+^ cDC2 and CD8α^+^ cDC1 from WT and CBL^-/-^CBL-B^-/-^ mice were purified by FACS sorting. Total RNAs from sorted cells (pooled from six mice) was extracted using an RNEasy Mini Kit (QIAGEN), and reversely transcribed into cDNA using a Reverse-Transcription kit (Invitrogen) according to manufacturer’s instructions, respectively. RNA-seq was performed using the Illumina TruSeq Stranded mRNA Kit according to manufacturer’s instructions on an Illumia HiSeq 2000 sequencer. Read quality was confirmed using FastQC v0.10.1. Read alignment was performed using TopHat v2.010 on the mouse GRCm38/mm10 genome. Differential expression analysis was performed with DESeq2 from the raw alignment counts calculated with featureCounts. For qPCR analysis, a SYBR Green PCR mix (Thermo Scientific) and gene-specific primers were used for quantitative RT-PCR analysis (20-50 ng cDNA per reaction). All reactions were done in triplicates with ViiA7-96 Real Time PCR System (Applied Biosystems). Results were analyzed by the change-in-threshold method, with β-Actin or GAPDH as ‘housekeeping’ reference genes.

### Rapamycin Blockade Assay

For *in vitro* rapamycin blocking assay, BM FLT3L culture (1:10 dilution of FLT3 conditional medium) was treated with different doses of rapamycin (LC Laboratories) starting at day 3. Generations of DC subsets were analyzed at day 7. For short-term *in vivo* rapamycin blockage, 10-week-old WT and CBL^-/-^CBL-B^-/-^ mice were injected i.p. with rapamycin (30 mg/day) for seven consecutive days. Mice were then sacrificed and splenic cDC subsets were examined by FACS. For long-term *in vivo* rapamycin blockade assay, 3-month-old RAG1.CBL tko mice were injected i.p. with PBS or rapamycin (100 mg/2 days). Mice were then monitored for the manifestation of inflammatory diseases.

### Statistical Analysis

Statistical analyses were performed with a two-tailed, unpaired Student’s t test or Fisher’s exact t test, with GraphPad Prism V7 software. A *P* value < 0.05 was considered statistically significant. For survival curve analysis, log-rank test was performed. A *P* value < 0.05 was considered statistically significant.

## Results

### The CBL^-/-^CBL-B^-/-^ Mutation Alters the Homeostasis of Peripheral CD8α^+^ cDC1

**Figure 1:**
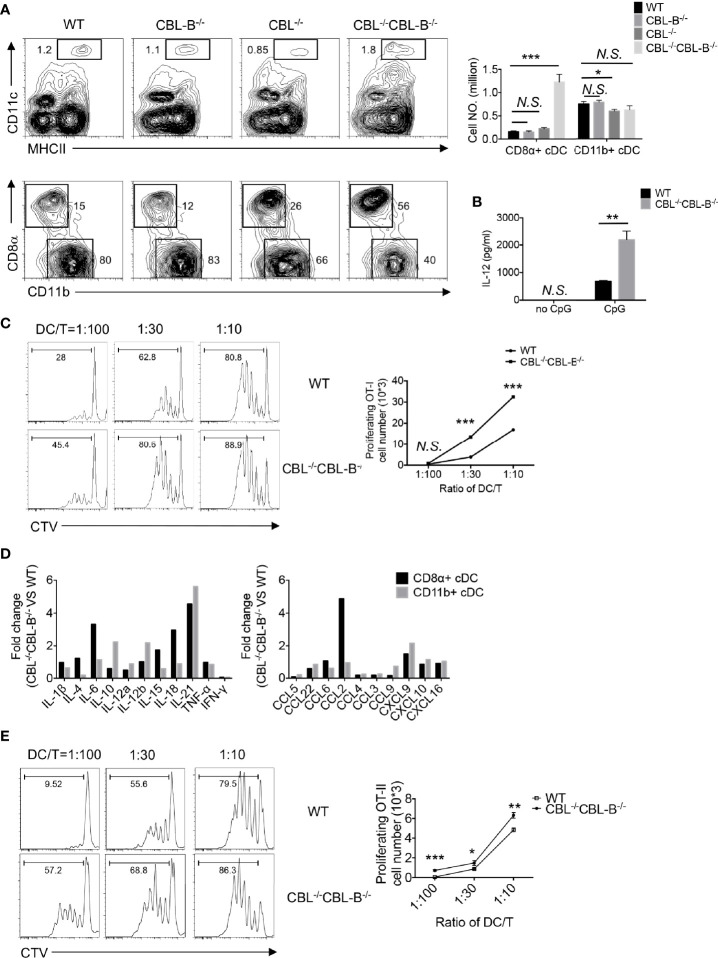
Analyses of the DC development in WT and CBL^-/-^CBLB^-/-^ mice. **(A)** Flow cytometry and statistical analyses of splenic cDCs. CD11c *vs* MHC-II staining of total splenic cDCs (Top panel), CD8α *vs* CD11b staining of CD8α^+^ cDC1 and CD11b^+^ cDC2 (bottom panel), and statistics (right bars) (n = 5). **(B)** IL-12 production by WT and CBL^-/-^CBL-B^-/-^ cDC1 upon CpG stimulation (n = 3). **(C)** Antigen cross presentation to CD8^+^ T cells by WT and CBL^-/-^CBL-B^-/-^ CD8α^+^ cDC1. Histograms of OT-I CD8^+^ T cell proliferation (left). Statistics of OT-I CD8^+^ T cell proliferation (right) (n = 3). **(D)** RNAseq analyses of cytokine and chemokine profiles of CD8α^+^ cDC1s and CD11b^+^ cDC2s. **(E)** Comparison of the antigen presentation efficiency of WT and CBL^-/-^CBL-B^-/-^ cDC1s to CD4^+^ T cells (n=3). Data are mean ± SEM. of at least two independent experiments **(A–E)**. N.S., not significant; *p < 0.05; **p < 0.01; ***p < 0.001.

In steady-state, peripheral DCs include cDC1s, cDC2s, and pDCs. To study the role of CBLs in DC development and function we generated DC specific CBL and CBL-B double null mutant mice by crossing *Cbl^flox/^* ^flox^ and *Cbl-b^-/-^* mice to *CD11c-Cre* transgenic (tg) mice in which DCs carried the *Cbl^-/-^* and *Cbl-b^-/-^ alleles* (termed the CBL^-/-^ CBL-B^-/-^ mutation), whereas all other lineages of cells harbored the germline *Cbl-b^-/-^* mutation (33, 34). Deletion of CBL and CBL-B in DCs was confirmed by Western blot analysis (**Supplementary Figures 1A, B**). Flow cytometry analysis revealed that the percentage of CD11c^+^ MHC-II^+^ cDCs in the spleen was increased approximately two folds in CBL^-/-^ CBL-B^-/-^ mice compared to WT, CBL^-/-^ or CBL-B^-/-^ mice (**Figure 1A**). While the subset of CD11b^+^ cDC2s remained unaltered, the total number of CD8α^+^ cDC1s increased fivefold in CBL^-/-^CBL-B^-/-^ mice relative to other control groups (**Figure 1A**). Splenic cDC1s in the mutant mice phenotypically resembled the canonic CD8α^+^ cDC1 rather than the alternative lineage of CD8α^+^ DC, as they expressed similar levels of cell surface CD8α, CD86 and PD-L1 (**Supplementary Figures 2A, B**) (36, 37). In addition, they were functionally consistent with the canonic cDC1s because they secreted a higher amount of IL-12 upon TLR stimulation and were able to cross-present soluble ovalbumin (Ova) antigen to OT-I TCR transgenic CD8^+^ T cells as efficiently as WT cDC1s (**Figures 1B, C**) (19). The total number of CD11c^low^ PDCA1^+^ pDCs was not affected by the CBL^-/-^CBL-B^-/-^ mutation in the bone marrow; however, they were significantly reduced in the spleen (**Supplementary Figures 2C, D**). The mutant pDCs failed to downmodulate chemokine receptor CXCR4 and upregulate CCR5, thus suggesting that CBLs may promote pDC migration to the periphery (**Supplementary Figure 2E**) (38, 39). Together these results demonstrate that CBLs selectively control the homeostasis of peripheral cDC1s and pDCs. Given that the development of cDC1s and pDCs was neither significantly altered in CBL^-/-^ nor in CBL-B^-/-^ mice, we conclude that it is a redundant function between CBL and CBL-B.

### CBL^-/-^CBL-B^-/-^ cDCs Exhibit an Altered Profile of Cytokine and Chemokine Expression

To study whether the CBL^-/-^CBL-B^-/-^ mutation affected the activation status of cDCs, we examined activation markers and gene transcription profiles of cDC1 and cDC2 subsets in young CBL^-/-^CBL-B^-/-^ mice before the onset of the inflammation. Both cDC1 and cDC2 from the mutant mice expressed similar amounts of MHC-II and conventional costimulatory ligands CD80, CD86, CD40 and PD-L1 compared to the corresponding subsets from WT, CBL^-/-^ and CBL-B^-/-^ mice (**Supplementary Figure 2B**). However, RNAseq analysis revealed that the mutant cDC1s constitutively produced higher amounts of IL-6, IL-18, and IL-21, and chemokines CCL2 and CXCL9, whereas mutant cDC2s expressed higher levels of IL-10, IL-12β, and IL-21 and chemokine CXCL9 relative to WT counterparts (**Figure 1D**, and **Supplementary Figure 3A**). The increased expression of IL-6 and CCL2 was further confirmed by the qPCR assay (**Supplementary Figure 3B**). To test whether mutant cDC1s were more potent at priming T cells, we co-cultured CFSE labeled OT-II CD4^+^ T cells with WT or CBL^-/-^CBL-B^-/-^ cDC1s in the presence of Ova antigen and then measured the proliferation of OT-II CD4^+^ T cells by FACS. The mutant cDC1s induced more vigorous proliferation of OT-II CD4^+^ T cells compared to WT cDC1s (**Figure 1E**). Together these results show that ablation of CBLs in DCs leads to the loss of the immune quiescent status of cDCs. However, the hyperactivation of the mutant cDCs appears to be atypical because it is not accompanied by the increased expression of conventional costimulatory ligands, but rather involves only the overexpression of several inflammatory cytokines and chemokines.

### The CBL^-/-^CBL-B^-/-^ Mutation Enhances the Proliferation but Differentially Affects the Survival of cDC1s and cDC2s

**Figure 2:**
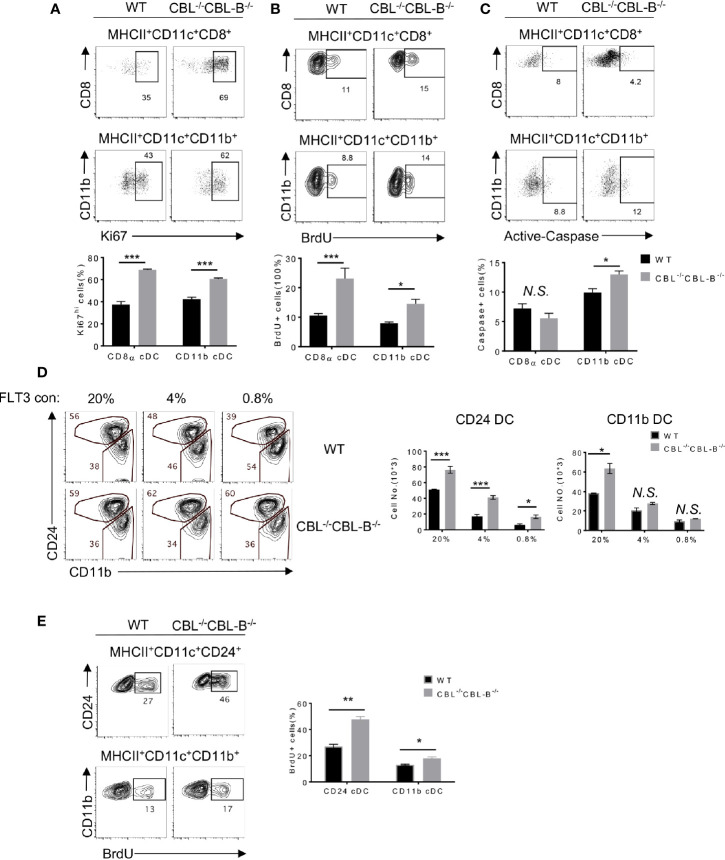
Proliferation, death, and lineage commitment of WT and CBL^-/-^CBL-B^-/-^ cDCs. **(A–C)** Flow cytometry (top panels) and statistics (bottom panels) of splenic proliferating **(A, B)** and apoptotic **(C)** CD8α^+^ cDC1s and CD11b^+^ cDC2s, as determined respectively by anti-Ki67 (n = 3) **(A)**, BrdU (n = 6) **(B)**, and anti-Active Caspase (n = 3) **(C)** staining. **(D)** Comparison of WT and CBL^-/-^CBL-B^-/-^ CD24^+^ (CD8α^+^-like) cDC1s and CD11b^+^ cDC2s in FLT3 dependent BM cell culture. Shown are the contour maps (left) and statistics (right) of CD24 *vs* CD11b staining of the gated CD11c^+^ cells (n = 3). **(E)** Proliferation of WT and CBL^-/-^CBL-B^-/-^ cDC generated in FLT3 dependent BM cell culture. Shown are FACS analyses (left) and statistics (right) of BrdU^+^ CD24^hi^CD11b^lo^ and CD24^lo^CD11b^hi^ cells (n = 3). Data are mean ± SEM. of at least two independent experiments. N.S., not significant; *p < 0.05; **p < 0.01; ***p < 0.001.

Homeostasis of cDC1s is influenced by lineage commitment of DC precursors as well as proliferation and survival of peripheral mature cDC1s. Since CD11c-Cre was mainly expressed in committed and mature DCs, we hypothesized that the CBL^-/-^CBL-B^-/-^ mutation affected proliferation and survival rather than lineage commitment of cDC1s. To test this hypothesis, we first examined the rate of DC proliferation based on the cell proliferation marker Ki67. We found that approximately 60-70% of splenic CBL^-/-^CBL-B^-/-^ cDC1s and cDC2s expressed a high amount of Ki67 (Ki67^hi^). In contrast, WT cDC1s and cDC2s contained only 40% of Ki67^hi^ cells (**Figure 2A**). The enhanced proliferation of the mutant cDC1s and cDC2s was confirmed by BrdU labeling as the mutant mice had approximately 50% more BrdU^+^ cDC1s and cDC2s relative to WT mice after overnight BrdU labeling (**Figure 2B**). In contrast to the increased proliferation, cDC1s in CBL^-/-^CBL-B^-/-^ mice appeared to have a slightly reduced population of cells expressing the active form of Caspase (Caspase^hi^) compared to WT counterparts (**Figure 2C**). In contrast, the mutant mice contained more Caspase^hi^ cDC2s relative to WT mice (**Figure 2C**), suggesting that the CBL^-/-^CBL-B^-/-^ mutation leads to a more profound apoptosis of cDC2s relative to cDC1s. Thus, the altered homeostasis of cDC1s but not cDC2s in CBL^-/-^CBL-B^-/-^ mice could be explained by a combined effect of enhanced proliferation of both mutant cDC1s and cDC2s and increased apoptosis of cDC2s.

Development of cDCs is driven by FLT3 signaling as well as the balanced action of transcription factors IRF8, IRF4, and ID2 (23). To examine whether these molecules were involved in the altered homeostasis of cDC1s in CBL^-/-^CBL-B^-/-^ mice, we analyzed DC development in FLT3-dependent bone marrow (BM) cell culture (40). CD8α^+^ cDC1-like cells and CD11b^+^ cDC2s were identified as MHC-II^+^ CD11c^+^ CD24^hi^ (CD24^hi^) and MHC-II^+^ CD11c^+^ CD11b^+^ CD24^low^ (CD11b^+^) cells, respectively. In the presence of a low concentration (0.8%) of FLT3L there were 50% more CD24^hi^ cDC1s derived from the CBL^-/-^CBL-B^-/-^ BM culture as compared to WT controls (**Figure 2D**). In contrast, the numbers of CD11b^+^ cDC2 generated in the mutant and WT BM cultures were comparable. The high concentration (20%) of FLT3L led to a more profound increase in CD24^hi^ DC1s in WT relative to the mutant BM cell culture, suggesting that the enhanced FLT3 signaling in WT cells may override the difference caused by the CBL^-/-^CBL-B^-/-^ mutation (**Figure 2D**). The increased generation of CD24^hi^ cDC1s in the mutant BM cell culture was likely a result of enhanced proliferation, because BrdU labeling analysis revealed more BrdU^+^ CD24^hi^ cDC1s and CD11b^+^ cDC2s in CBL^-/-^CBL-B^-/-^ BM culture compared to WT controls (**Figure 2E**). To examine whether the CBL^-/-^CBL-B^-/-^ mutation affected the expression of cDC1 lineage commitment genes, we purified CD24^hi^ cDC1s and CD11b^+^ cDC2s from BM cell FLT3L culture and examined IRF4 and IRF8 expression by Western blot analysis. Consistent with RNAseq data, the expression of these transcription factors was comparable between the WT and mutant cDC subsets (**Supplementary Figures 3A, C**). These results together support that CBLs regulate the homeostasis of cDC1s by controlling the proliferation rather than the lineage commitment of cDC1s through modulating FLT3 signaling.

### CBLs Control FLT3-AKT Signaling by Promoting FLT3 Ubiquitination

**Figure 3:**
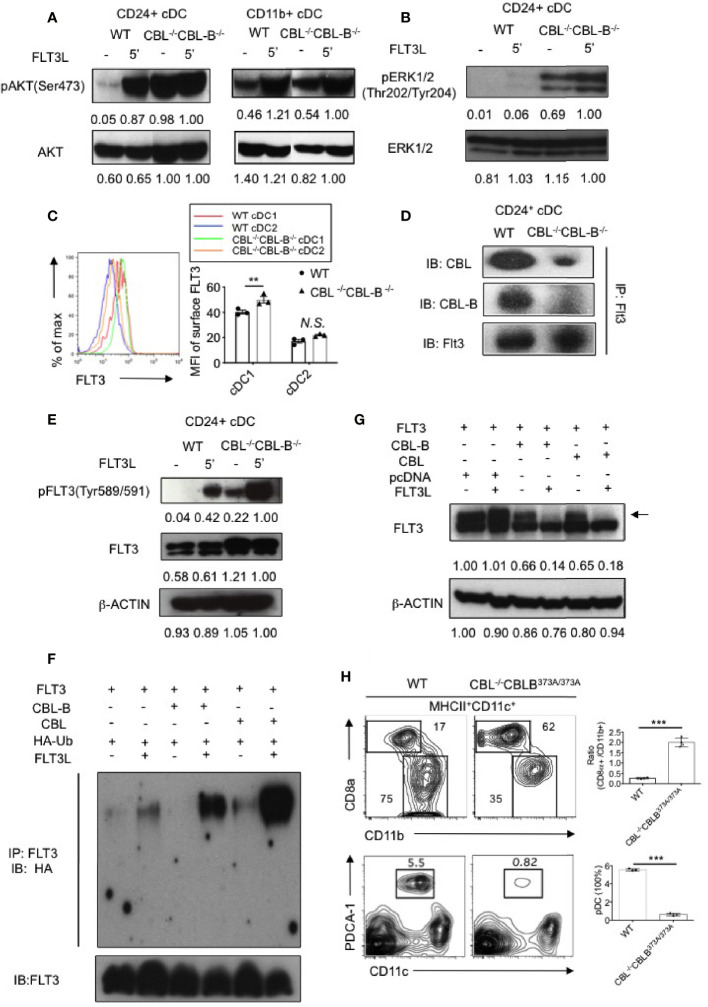
Analyses of FLT3 ubiquitination and signaling by CBLs. **(A)** Western blot analysis of AKT activation in WT and CBL^-/-^CBL-B^-/-^ cDC1s and cDC2s (n = 2). **(B)** Western blot analysis of ERK1/2 activation in WT and CBL^-/-^CBL-B^-/-^ cDC1s (n = 2). **(C)** Cell surface expression of FLT3 in WT and CBL^-/-^CBL-B^-/-^ cDC1s and cDC2s (n = 3). **(D)** Association of FLT3 with CBL and CBL-B in BM derived CD11c^+^CD24^hi^ cDC1s (n = 2). **(E)** Western blot analysis of FLT3 activation. pFLT3 (Tyr589/591): active form of FLT3 (n = 2). **(F)** Western blot analysis of FLT3 ubiquitination (n = 2). **(G)** Western blot analysis of phosphorylated FLT3 (pFLT3) (n = 2). **(H)** Dependence of CD8α^+^ cDC1 and pDC homeostasis on CBL ubiquitin ligase activity. Shown are contour maps (left) and statistics (right) of splenic cDC1s and cDC2s (top) and pDC (bottom) in WT and CBL^-/-^CBL-B^C373A/C373A^ mice (n = 3-4). Data are mean ± SEM. of at least two independent experiments **(A**, **B**, **D**–**H)**, and one experiment for **(C)** N.S., not significant; **p < 0.01; ***p < 0.001.

FLT3 is a receptor tyrosine kinase that transduces signals through both the MAP kinase and PI3 kinase pathways, the latter activating AKT-mTOR signaling. We therefore examined whether the CBL^-/-^CBL-B^-/-^ mutation enhanced PI3 kinase and MAP kinase signaling. Since we could not obtain enough splenic cDCs for a biochemical study, we used BM cell derived CD24^hi^ cDC1s and CD11b^+^ cDC2s in our experiments. In unstimulated WT CD24^hi^ cDC1s, the AKT activity was minimal and stimulation of FLT3 significantly increased AKT activity (**Figure 3A**). In contrast, CBL^-/-^CBL-B^-/-^ CD24^hi^ cDC1s showed a very high level of constitutive AKT activity that may be maximal since FLT3 stimulation did not further enhances the signal. Similar to AKT activation, unstimulated CBL^-/-^CBL-B^-/-^ CD24^hi^ cDC1s contained elevated active forms of ERK1/2 and stimulation of FLT3 elicited even greater ERK1/2 activity in the mutant cells compared to WT controls (**Figure 3B**). In CD11b^+^ cDC2s, FLT3L stimulation elicited a comparable level of AKT activity between WT and the mutant cells, suggesting that CBLs exert little effect on FLT3 signaling in these cells (**Figure 3A**). These findings thus suggest that CBLs directly target FLT3 in CD24^hi^ cDC1s.

Since CBLs are E3 ubiquitin ligases, we next examined whether CBLs negatively regulate FLT3 signaling by promoting FLT3 ubiquitination and degradation. Flow cytometric analysis revealed that FLT3 expression was higher on WT CD24^hi^ cDC1s compared to that on cDC2s (**Figure 3C**). In CD24^hi^ cDC1s, both CBL and CBL-B were associated with FLT3 (**Figure 3D**). Ablation of CBLs markedly increased the active form of FLT3 (pFLT3 Tyr^589/591^) in CD24^hi^ cDC1s (**Figure 3E**), supporting the idea that CBLs promote the clearance of the active form of FLT3. To determine whether CBLs promote FLT3 ubiquitination, we co-expressed FLT3 with CBL or CBL-B in 293T cells and examined FLT3 ubiquitination with or without FLT3L stimulation. Expression of either CBL or CBL-B promoted FLT3 ubiquitination upon FLT3L stimulation (**Figure 3F**). Consistent with this finding the expression of either CBL or CBL-B in 293T cells removed the active form of FLT3 (**Figure 3G**).

To determine whether the altered homeostasis of CD8α^+^ cDC1 was related to CBL mediated ubiquitination, we examined CD8α^+^ cDC1 and CD11b^+^ cDC2 development in DC specific CBL^-/-^ CBL-B^C373A/C373A^ mice. The mutant CBL-B^C373A/C373A^ mice carried a cysteine (373) to alanine mutation in CBL-B that inactivates only the ubiquitin ligase activity but does not affect the scaffolding function of CBL-B (35). Compared to WT mice, CBL^-/-^ CBL-B^C373A/C373A^ mice possessed a similarly increased number of cDC1s and a reduced number of pDCs as that found in CBL^-/-^CBL-B^-/-^ mice (**Figure 3H**). This finding indicates that inactivation of the ubiquitin ligase activity of CBLs is fully responsible for the altered DC homeostasis in CBL^-/-^CBL-B^-/-^ mice.

Together we propose that CBLs control the strength of FLT3 signaling by promoting FLT3 ubiquitination. The preferential expression of FLT3 in CD8α^+^ cDC1s as compared to CD11b^+^ cDC2s could explain why the CBL^-/-^CBL-B^-/-^ mutation exerts a more profound effect on the homeostasis of cDC1s relative to cDC2s.

### Ablation of CBLs in DCs Leads to Manifestation of Severe Liver Inflammatory Disease and Early Fatality

**Figure 4:**
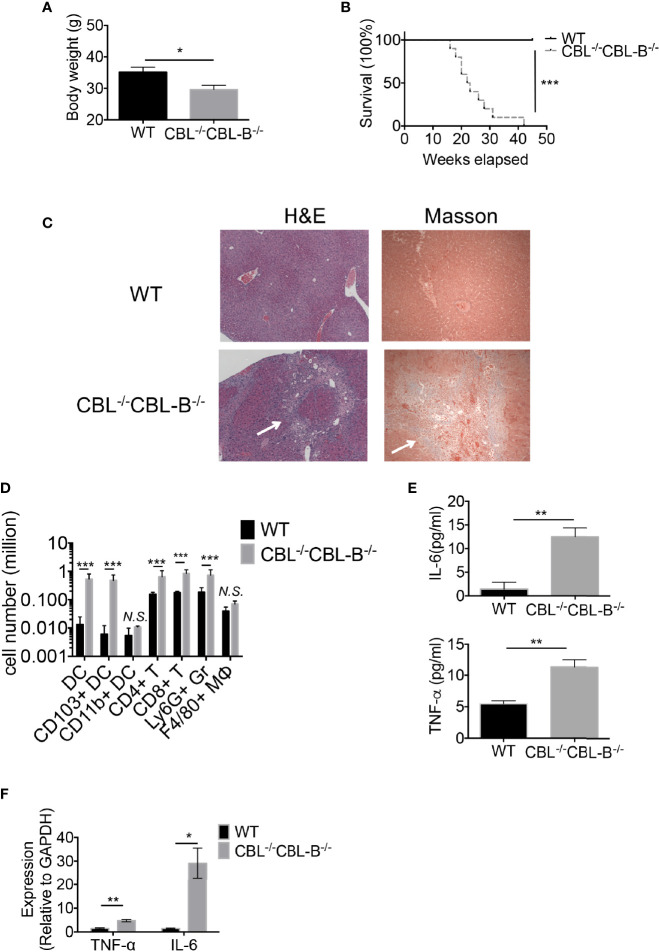
CBL^-/-^CBL-B^-/-^ mice have a reduced lifespan and severe inflammation. **(A)** Body weight comparison between 12-week-old WT and CBL^-/-^CBL-B^-/-^ mice (n = 5). **(B)** Kaplan-Meier survival analysis of WT (n = 20) and CBL^-/-^CBL-B^-/-^ (n = 20) mice. **(C)** Pathological analysis WT and CBL^-/-^CBL-B^-/-^ mice. Shown are H-E staining and Masson staining of the liver. Infiltrating leukocytes and liver fibrosis are indicated by arrows. **(D)** Flow cytometric analysis of liver leukocyte subsets. Shown are the statistics of infiltrated cell subsets of the gated CD45^+^ cells in livers from WT and CBL^-/-^CBL-B^-/-^ mice (n = 5). DC: CD11c^+^MHC-II^hi^; CD103^+^ DC: CD11c^+^MHC-II^hi^CD103^+^CD8α^+^; CD11b^+^ DC: CD11c^+^MHC-II^hi^CD11b^+^; CD4^+^ T: TCRβ^+^CD4^+^; CD8^+^ T: TCRβ^+^CD8^+^; Ly6G^+^ Gr: CD11b^+^Ly6G^+^; F4/80^+^ MΦ: CD11b^+^F4/80^+^. **(E)** Cytokine IL-6 and TNF-α secretion in serum (n = 5-6). **(F)** qPCR analysis of cytokine IL-6 and TNF-α expression in the liver (n = 3). Data are mean ± SEM. of at least two independent experiments **(C**–**F)**. N.S., not significant; *p < 0.05; **p < 0.01; ***p < 0.001.

Increased numbers and hyperactivation status of cDCs in CBL dko mice prompted us to examine whether the CBL^-/-^CBL-B^-/-^ mutation impaired immune tolerance and caused autoimmune diseases. Our inspection revealed that CBL^-/-^CBL-B^-/-^ mice were healthy at birth; however, they gradually lost bodyweight and became moribund before six months of age (**Figures 4A, B**). Pathological studies revealed that the mutant mice developed exclusively severe liver inflammatory diseases, characterized by serum and skin jaundice, massive liver infiltration of leukocytes, and liver infarct and fibrosis (**Figure 4C**). In a small number of mutant mice, widespread infiltration of leukocytes was also found in other tissues such as the lung and kidney (**Supplementary Figure 4A**). While spleen architecture of the mutant mice was significantly distorted, we did not detect serum auto-antibodies against double-strained DNA or nuclear antigens, suggesting that B cells are probably not involved in the manifestation of the tissue inflammation.

Given that the mutant mice developed severe liver inflammation, we examined the cellularity of liver infiltrating cells by flow cytometry and cytokine production by qPCR and ELISA. We found that livers from the diseased mutant mice contained markedly increased numbers of CD103^+^ cDC1s, CD4^+^ and CD8^+^ T cells, and Ly6G^+^ granulocytes (**Figure 4D**). Consistent with the severe liver inflammation, the mutant mice produced significantly higher amounts of serum cytokines IL-6, TNFα and inflammatory chemokines relative to WT controls (**Figure 4E**). Compared to WT counterparts the mutant liver also produced high levels of IL-6 and TNF-α, indicating that the liver has hyper inflammation (**Figure 4F**). In contrast, the total number of liver F4/80^+^ macrophages was not increased (**Figure 4D**), suggesting that Kupffer cells and recruited macrophages are not the major players responsible for the disease.

### DC Intrinsic CBLs Are Required for the Maintenance of T Cell Immune Quiescence in Steady-State

**Figure 5:**
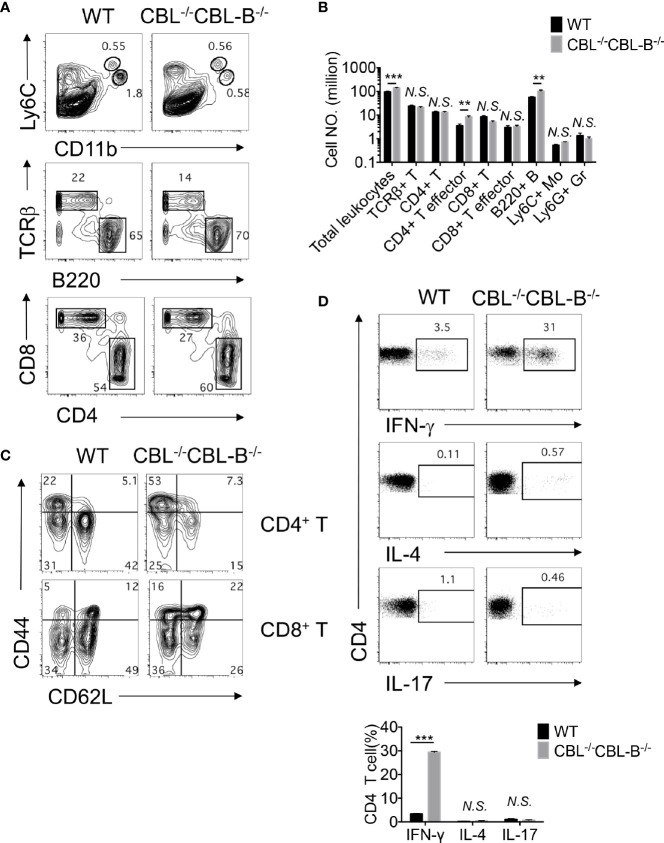
Impaired immune quiescence in CBL^-/-^CBL-B^-/-^ mice. **(A, B)** Flow cytometry and statistical analyses of the spleen cellularity (n = 5). **(C)** Activation status of splenic CD4^+^ and CD8^+^ T cells. Shown are contour maps of CD44 *vs* CD62L staining of naïve (CD44^lo^CD62L^hi^), memory/effector (CD44^hi^CD62L^hi^), effector (CD44^hi^CD62^lo^) T cells (n = 5). **(D)** T effector T cell subsets in the spleen. Th1, Th2, and Th17 cell subsets were stained by anti-TCRβ, CD4, and intracellular IFN-γ, IL-4, and IL-17, respectively (n = 3). Data are mean ± SEM. of at least two independent experiments **(A–D)**. N.S., not significant; **p < 0.01; ***p < 0.001.

Development of spontaneous liver inflammation prompted us to examine whether CBL^-/-^CBL-B^-/-^ DCs elicited systemic hyperactivation of the immune system. We found that before the disease development CBL^-/-^CBL-B^-/-^ mice possessed similar numbers of splenic CD4^+^ and CD8^+^ T cells, Ly6G^+^ granulocytes, and Ly6C^+^ monocytes as compared to WT mice (**Figures 5A, B**). However, compared to WT mice, significantly increased numbers of CD4^+^ and CD8^+^ T cells in the mutant mice exhibited the phenotype of effector/memory T cells as they expressed a higher amount of CD44 and reduced CD62L (**Figure 5C**). In addition, a large proportion of activated CD4^+^ T cells in the mutant mice adopted the T_H1_ cell fate as they secreted a higher amount of IFN-γ but not IL-4 nor IL-17 upon activation (**Figure 5D**). This finding thus indicates that under steady-state CBLs in CD11c^+^ cells including cDCs are responsible for the maintenance of immune quiescence of CD4^+^ and CD8^+^ T cells.

### Manifestation of Liver Inflammation in CBL ^-/-^CBL-B^-/-^ Mice Is Independent of T and B Cells

**Figure 6:**
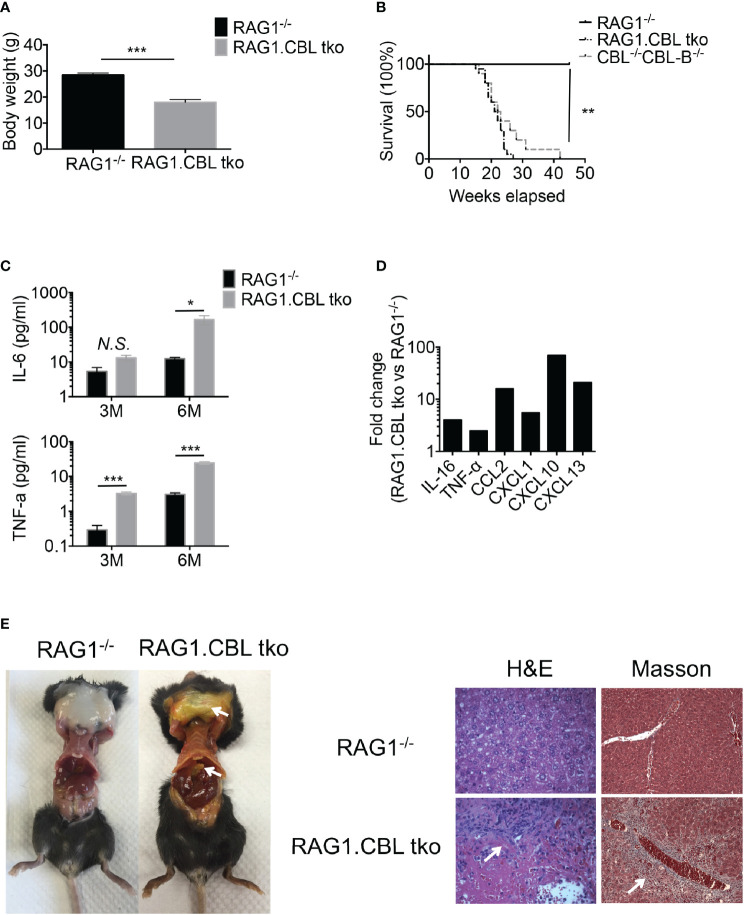
Development of liver inflammation and fibrosis in CBL^-/-^CBL-B^-/-^ mice is independent of lymphocytes. **(A)** Body weight comparison between 12-week-old RAG1^-/-^ and RAG1.CBL tko mice (n = 5). **(B)** Kaplan-Meier survival analysis of RAG1^-/-^, CBL^-/-^CBL-B^-/-^, and RAG1^-/-^CBL tko mice (n = 20). **(C)** Serum Cytokines IL-6 and TNF-α production in RAG1^-/-^ and RAG1.CBL tko mice by ELISA (n = 3-4). **(D)** Serum inflammatory cytokines and chemokines in RAG1.CBL tko mice. **(E)** Images of skin jaundice (left) and histology analysis of liver fibrosis revealed by H-E staining (right panel) and Masson staining (right panel). Data are mean ± SEM. of at least two independent experiments **(C–E)**. N.S., not significant; *p < 0.05; **p < 0.01; ***p < 0.001.

Hyperactivation of T cells in CBL^-/-^CBL-B^-/-^ mice suggested that the observed liver inflammation was caused by T cells activated by CBL^-/-^CBL-B^-/-^ DCs. To assess the contribution of T cells and CBL^-/-^CBL-B^-/-^ DCs in disease development we monitored RAG1^-/-^CBL^-/-^CBL-B^-/-^ triple knockout (RAG1.CBL tko) mice that lack T and B cells. The RAG1.CBL tko mice quickly lost body weight relative to RAG1^-/-^ littermates and had a shorter lifespan even compared to CBL^-/-^CBL-B^-/-^ mice (**Figures 6A, B**). Analyses of cytokine and chemokine profiles showed that RAG1.CBL tko mice produced higher amounts of serum IL-6 and TNF-α and inflammatory chemokines such as CCL2, CXCL1, CXCL10, and CXCL13 compared to RAG1^-/-^ controls (**Figures 6C, D**, and **Supplementary Figure 4B**). RAG1.CBL tko mice also manifested exclusively liver inflammation, characterized by skin jaundice, massive liver infiltration of leukocytes and fibrosis (**Figure 6E**), similar to that found in CBL^-/-^CBL-B^-/-^ mice. Flow cytometric analysis of liver infiltrating cells showed a ten-fold increase in CD103^+^ cDC1s, whereas numbers of liver infiltrating NK1.1^+^ cells and F4/80 macrophages^+^ were not increased or slightly reduced (**Supplementary Figure 4C**). Thus, in CBL^-/-^CBL-B^-/-^ mice while T cells are massively activated, manifestation of liver inflammation is positively correlated with the increased CD103^+^ cDC1s but is not dependent on T and B cells.

### Blockade of mTOR Signaling in CBL^-/-^CBL-B^-/-^ Mice Restores the Homeostasis of cDC1s and Immune Quiescence and Attenuates Liver Inflammation

**Figure 7:**
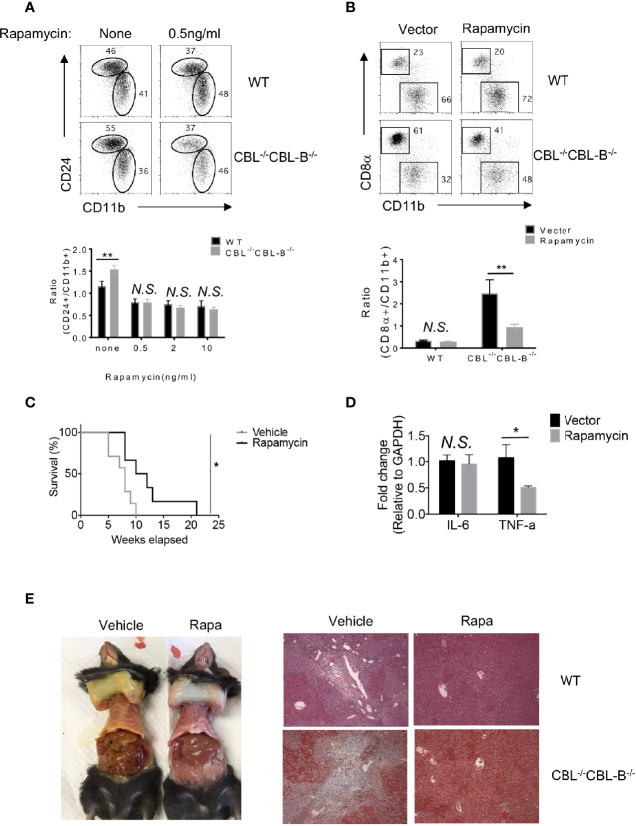
Homeostasis of cDC1s and liver inflammation in mTOR treated CBL^-/-^CBL-B^-/-^ mice. **(A)** Flow cytometry analysis of CD24^hi^CD11b^lo^ and CD24^lo^CD11b^hi^ cDCs in FLT3-dependent BM culture treated with rapamycin (n = 3). **(B)** Flow cytometry analysis of CD8α^+^ cDC1s and CD11b^+^ cDC2s in WT and CBL^-/-^ CBL-B^-/-^ mice with or without rapamycin treatment (n = 3). **(C)** Kaplan-Meier survival analysis of CBL^-/-^CBL-B^-/-^ mice treated with rapamycin or vehicle (n = 6-7). **(D)** qPCR analysis of liver IL-6 and TNF-α in rapamycin treated CBL^-/-^CBL-B^-/-^ mice (n = 5). **(E)** Pathology analysis of CBL^-/-^CBL-B^-/-^ mice with rapamycin or vehicle treatment. Left: Image of skin jaundice. Right: H-E staining of liver sections. Data are mean ± SEM. of at least two independent experiments **(A**, **B**, **D**, **E)**. N.S., not significant; *p < 0.05; **p < 0.01.

Enhanced FLT3-AKT signaling in CBL^-/-^ CBL-B^-/-^ cDC1s suggests that attenuation of FLT3-AKT signaling may normalize cDC1 homeostasis and cure liver inflammatory disease in CBL^-/-^CBL-B^-/-^ mice. Since AKT activates mTORs, we examined whether blockade of mTOR signaling reduced the number of cDC1s to normality and restored immune quiescence in CBL^-/-^CBL-B^-/-^ mice. To do so, we pharmacologically blocked mTOR signaling using rapamycin both *in vitro* and in mice and then analyzed cDC development by flow cytometry. We found that rapamycin treatment significantly diminished the number of CD24^hi^ cDC1s in CBL^-/-^CBL-B^-/-^ BM cell FLT3L culture compared to WT controls (**Figure 7A**). Consistent with this finding, *in vivo* rapamycin treatment significantly reduced the number of cDC1s in CBL^-/-^CBL-B^-/-^ mice (**Figure 7B**). In contrast, the same rapamycin treatment exhibited little effect on the numbers of CD11b^+^ cDC2s in both BM cell FLT3L culture and mice, indicating that mTOR signaling selectively regulates the development of cDC1s. To examine whether blockade of mTOR signaling influenced immune quiescence and liver inflammation, we monitored the survival and pathological changes of rapamycin treated CBL^-/-^CBL-B^-/-^ mice. Compared to PBS treated CBL^-/-^ CBL-B^-/-^ mice, rapamycin treated mutant mice had significant longer lifespans (**Figure 7C**). In addition, they had markedly reduced levels of TNF-α in the liver and absence of liver inflammation and fibrosis compared to the PBS treated cohort, possibly due to reduced infiltration of inflammatory cells (**Figures 7D, E**). These results thus support the notion that enhanced FLT3-mTOR signaling in CBL^-/-^CBL-B^-/-^ DCs is responsible for the altered homeostasis of cDC1s. In addition, they provide evidence that this signaling axis is at least partly responsible for the loss of immune quiescence and the development of lethal liver inflammation and fibrosis in the mutant mice.

## Discussion

Under steady-state, DC function is maintained quiescent. However, since microbial and immune danger signals are constantly present, there must be mechanisms that restrain DCs from overreaction. Loss of DC immune quiescence has been found in the mutant mouse models such as those deficient in the negative regulators for TLRs, NFκB, and PI3 kinases (12, 13, 25). In addition, it has been shown that depletion of DCs or increase in DC lifespan leads to myeloid lineage expansion or autoimmunity (14, 15), suggesting that the homeostasis of DCs also influences the functional quiescence of DCs. In this report, we show that ablation of CBLs in DCs alters DC homeostasis, with a marked increase in the number of CD8α^+^ and liver CD103^+^ cDC1s, and a reduction in peripheral pDCs. CBL^-/-^CBL-B^-/-^ mice manifest spontaneous liver inflammatory diseases and fibrosis. Treatment of the mutant mice with rapamycin reduces the number of cDC1s and attenuates the liver inflammation. Our finding thus identifies CBLs as critical regulators to maintain peripheral DC homeostasis and supports that altered DC homeostasis may breakdown immune quiescence under steady-state. In addition, we also provide evidence that modulation of DC homeostasis can be an effective approach to treat liver inflammation.

How do CBLs in DCs regulate immune quiescence and prevent liver inflammation at steady-state? Our results reveal that CBL^-/-^CBL-B^-/-^ mice have a great expansion of the cDC1 population. While the mutant cDCs do not exhibit typical activation phenotypes, such as upregulation of cell surface costimulatory ligands CD80, CD86, and MHC-II, freshly isolated mutant cCD1s and cDC2s produce slightly more IL-6 and significantly higher levels of inflammatory chemokines, even in the absence of stimulation. Since CD103^+^ cDC1s are significantly increased in the liver, we propose that the increased number of cDC1s, and the amount of inflammatory cytokines and chemokines therein, create an inflammatory environment that recruits granulocytes and other inflammatory cells to the liver, leading to the development of liver inflammation. At present, it is not clear whether microbial and other danger signals such as those released by dead cells contribute to the activation of the liver CD103^+^ cDC1s. However, since CpG stimulation enhances the production of IL-6 and chemokines in CBL^-/-^CBL-B^-/-^ cDCs relative to WT counterparts, we speculate that microbial products or self danger cues in the liver might also be involved in disease development by triggering DC activation *de novo via* TLRs. Consistent with this prediction, we have found that CBL^-/-^CBL-B^-/-^ mice deficient in MyD88 (termed as MyD88^-/-^.CBL tko mice), while still have a similarly increased number of CD8α^+^ cDC1s, exhibit much longer lifespan and less severe liver inflammation relative to CBL^-/-^CBL-B^-/-^ mice (**Supplementary Figures 5A, B**). Thus, in addition to the homeostasis of cDC1s, the CBL^-/-^CBL-B^-/-^ mutation might also alter MyD88 dependent signaling. It will be interesting to further elucidate which TLRs are regulated by CBLs. It will also be important to determine whether blockade of both mTOR and MyD88 completely prevents development liver inflammation.

The development of cDCs depends on FLT3 signaling and our finding that CBLs negatively regulate FLT3 signaling by promoting FLT3 ubiquitination and degradation is in agreement with the previous finding that enhanced FLT3-PI3K activity in DCs leads to increased number of cDC1s (25, 36). However, we have shown that the total number of cDC2 is not increased and the number of splenic pDCs is even reduced in the mutant mice, suggesting that other regulations may also exert a role in DC subset homeostasis. Indeed, our data shows that while cDC2s from CBL^-/-^CBL-B^-/-^ mice also proliferate more vigorously relative to WT counterparts, they are more prone to apoptosis than the mutant cDC1s. The reason behind such an alteration is not yet clear. One plausible explanation is that cDC2s are less competitive to access the limited amount of FLT3L available in periphery, because they express relatively lower amount of FLT3 as compared to cDC1s. As for the reduced number of pDCs, our data indicates that they are generated normally in the BM, however, markedly reduced in the periphery. Gene expression profile reveals that CBL^-/-^CBL-B^-/-^ pDCs fail to downmodulate CXCR4 and upregulate CCR5. Given that CXCR4 downmodulation and CCR5 upregulation have been linked to pDC migration from the BM to periphery (39), our finding thus suggests that CBLs regulate the exit of pDCs to periphery rather than the overall development of pDC in the BM.

Currently, cellular mechanisms leading to liver fibrosis are not fully understood. Inflammation following liver damage is considered to be one of the main causes triggering the cascade of hepatic stellate cell activation and liver fibrosis (26). Kupffer cells and recruited macrophages have been shown to play a major role in liver inflammation (29). While evidence implicates that Th17 cells could also be involved in promoting liver inflammation and fibrosis by stimulating Kupffer cells and macrophages to produce inflammatory cytokines and hepatic stellate cells to produce collagen type-I and differentiate to fibrogenic myofibroblasts (41, 42), liver DCs are generally considered tolerogenic and their role in the pathology of liver inflammation and fibrosis has not been extensively explored, despite the fact that extrahepatic DCs have been shown to affect liver inflammation (26, 43). While our data cannot completely exclude the possibility that CBLs were deleted in some macrophages/Kupffer cells or NK cells due to the potential leakiness of CD11c-Cre tg expression, the CBL mutant macrophages are less likely a major cause to induce liver inflammation because we have found that the number of liver macrophages is not increased and cytokine production of BM-derived CBL macrophages from CBL^-/-^CBL-B^-/-^ mice is not altered. In addition, ablation of CBL and CBL-B in monocytes and macrophages using the Lys-Cre allele does not cause liver inflammation in mice (44). In contrast, our data reveal a close correlation between the number and inflammatory cytokine and chemokine production of liver CD103^+^ cDC1s and inflammation and fibrosis. Blockade of mTOR signaling by rapamycin in CBL^-/-^ CBL-B^-/-^ mice concomitantly reduces the number of liver CD103^+^ cDC1s and the incidence of liver inflammation and fibrosis. In this regard, data from our mutant mice supports a further study to elucidate whether DCs are also involved in the liver inflammation and fibrosis in humans.

## Data Availability Statement

The datasets presented in this study can be found in online repositories. The names of the repository/repositories and accession number(s) can be found below: https://osf.io/2kst3/?view_only=53c4e1cc99e84069af0c7c6db21bcaf6.

## Ethics Statement

The animal study was reviewed and approved by The Institut de Recherches Cliniques de Montréal (IRCM) Animal Care Committee.

## Author Contributions

HT, XL, and JZ did mouse, biochemical, and flow cytometric analyses. LG, WS, XZ, AG, and YL conducted some mouse and *in vitro* cell culture experiments. VC contributed to bioinformatics analysis. BR provided CD-11c Cre tg mice and B16-FLT3L cell line and contributed to manuscript preparation. WL provided CBL^C373A/C373A^ mice. YZ contributed to the design of the experiments and manuscript preparation. HG is responsible for the overall designs of the experiment and manuscript writing. All authors contributed to the article and approved the submitted version.

## Funding

This work was Supported by The A. Aisenstadt Chair Fund to HG; Chinese Scholarship Council Ph.D. training grants to HT and XL. Grants from The National Natural Science Foundation of China (31270939, 81471526, 81771667), Training Program of the Major Research Plan in regional immunology of the National Natural Science Foundation of China (91442110), and PCSIRT (IRT1075) to JZ; National Health and Medical Research Concil project grant (1101318) and the Medical and Health Infrastructure Fund to WL, and a NSHLIJ Institutional Fund to YZ.

## Conflict of Interest

The authors declare that the research was conducted in the absence of any commercial or financial relationships that could be construed as a potential conflict of interest.

## Publisher’s Note

All claims expressed in this article are solely those of the authors and do not necessarily represent those of their affiliated organizations, or those of the publisher, the editors and the reviewers. Any product that may be evaluated in this article, or claim that may be made by its manufacturer, is not guaranteed or endorsed by the publisher.

## Supplementary Material

Supplementary Figure 1 Expression of CBL and CBL-B in DC and lymphocyte subsets and pathological analysis of CBL-/-CBL-B-/- mice (A) FACS sorting strategy for purifying splenic pDCs, cDCs, T and B cells. (B) Western blot analysis of CBL and CBL-B expression in pDCs, cDCs, T and B cells. Data represents one of two experiments.

Supplementary Figure 2 Characterization of CD8a+ cDC1s and pDCs **(A)** CD8a *vs* CD11b staining of gated CD11c+ MHC-II+ splenic cDCs in WT and CBL-/-CBL-B-/- mice. The experiment was repeated for more than three times. **(B)** Expression of costimulatory ligands on CD8a+ cDC1s and CD11b+ cDC2s. The experiment was repeated for more than three times. **(C, D)** FACS analysis of the bone marrow (BM) and splenic (SP) pDCs. Shown are PDCA-1 vs CD11c staining of BM and spleen cells **(C)** and statistics **(D)**. (n = 5). (E) qPCR analysis of CXCR4 and CCR5 expression in WT and CBL-/-CBL-B-/- pDCs. (n = 3). Data are means ± SEM of at least five mice or three independent experiments. *p < 0.01; **p < 0.001.

Supplementary Figure 3 Gene and protein expression profiles in WT and CBL-/-CBL-B-/- cDC1s and cDC2s **(A)** Heatmap analysis of the chemokines, cytokines and genes related to DC development and function. Data represent pooled RNA samples from 6x WT and 6x CBL-/-CBL-B-/- mice. **(B)** qPCR analysis of TNF-a, IL-6 and CCL2 expression in WT and CBL-/-CBL-B-/- CD8a+ cDC1s. Shown are fold increases of the corresponding gene transcripts in the mutant cells relative to WT cells. (n = 3). Data are means ± SEM of at least three independent experiments. *p < 0.01. **(C)** Western blot analyses of IRF4 and IRF8 in WT and CBL-/-CBL-B-/- CD24hi cDCs.

Supplementary Figure 4 Pathological analyses of CBL-/-CBL-B-/- mice and RAG1.CBL tko mice **(A)** H&E staining of lung and kidney sections of WT and sick CBL-/-CBL-B-/- mice. Data are from one of five mice. **(B)** Serum cytokine and chemokine titers. Shown are dot blot hybridization of serum cytokine and chemokine titers of pooled serum samples from WT RAG1-/- and sick RAG1.CBL tko mice. (n = 3). **(C)** Total numbers of inflammatory cell subsets in liver infiltrating leukocytes of RAG1-/- and sick RAG1.CBL tko mice. (n = 5). Data are means ± SEM of at least five mice. *p < 0.01; **p < 0.001.

Supplementary Figure 5 Flow cytometric analysis of cDC subsets in and lifespan analysis of MyD88.CBL tko mice **(A)** FACS analysis (left) and statistics (right) of cDCs and CD8a+ cDC1s and CD11b+ cDC2s in WT (C57BL/6 or MyD88-/-) and MyD88.CBL tko mice. (n = 6). **(B)** Kaplan Meier survival analysis of WT (MyD88-/-), CBL-/-CBL-B-/- and MyD88.CBL tko mice. (n=10). Data are mean ± SEM of two independent experiments. *P < 0.01; **p < 0.001.
