# Supplementary figures and images for: Ubiquitin Ligases CBL and CBL-B Maintain the Homeostasis and Immune Quiescence of Dendritic Cells
Source: Front Immunol. 2021 Sep 23;12:757231. doi: 10.3389/fimmu.2021.757231 (PMC8494778; doi:10.3389/fimmu.2021.757231)

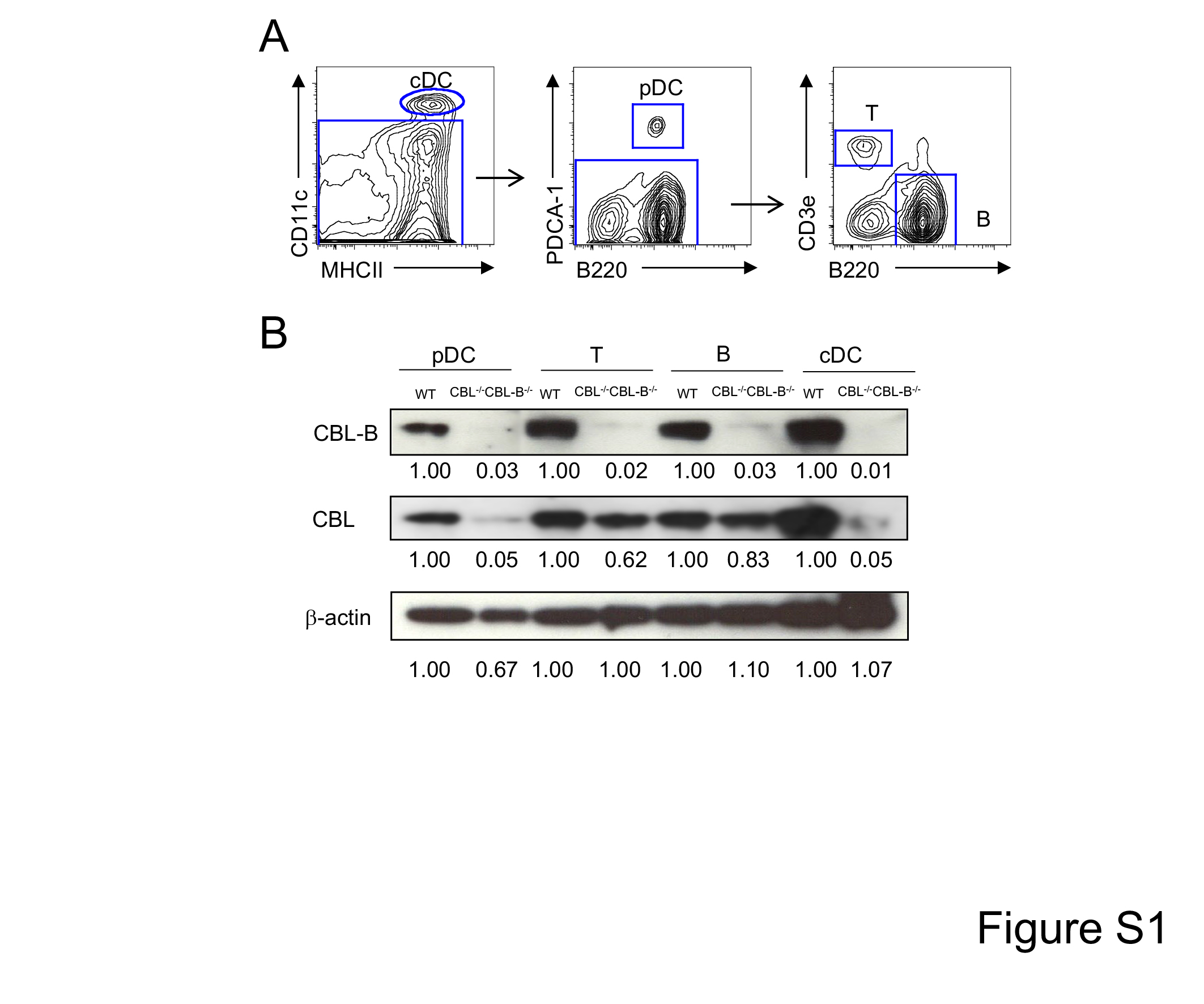

Supplement: Supplementary Figure 1 — Expression of CBL and CBL-B in DC and lymphocyte subsets and pathological analysis of CBL-/-CBL-B-/- mice (A) FACS sorting strategy for purifying splenic pDCs, cDCs, T and B cells. (B) Western blot analysis of CBL and CBL-B expression in pDCs, cDCs, T and B cells. Data represents one of two experiments. [file Image_1.jpeg]

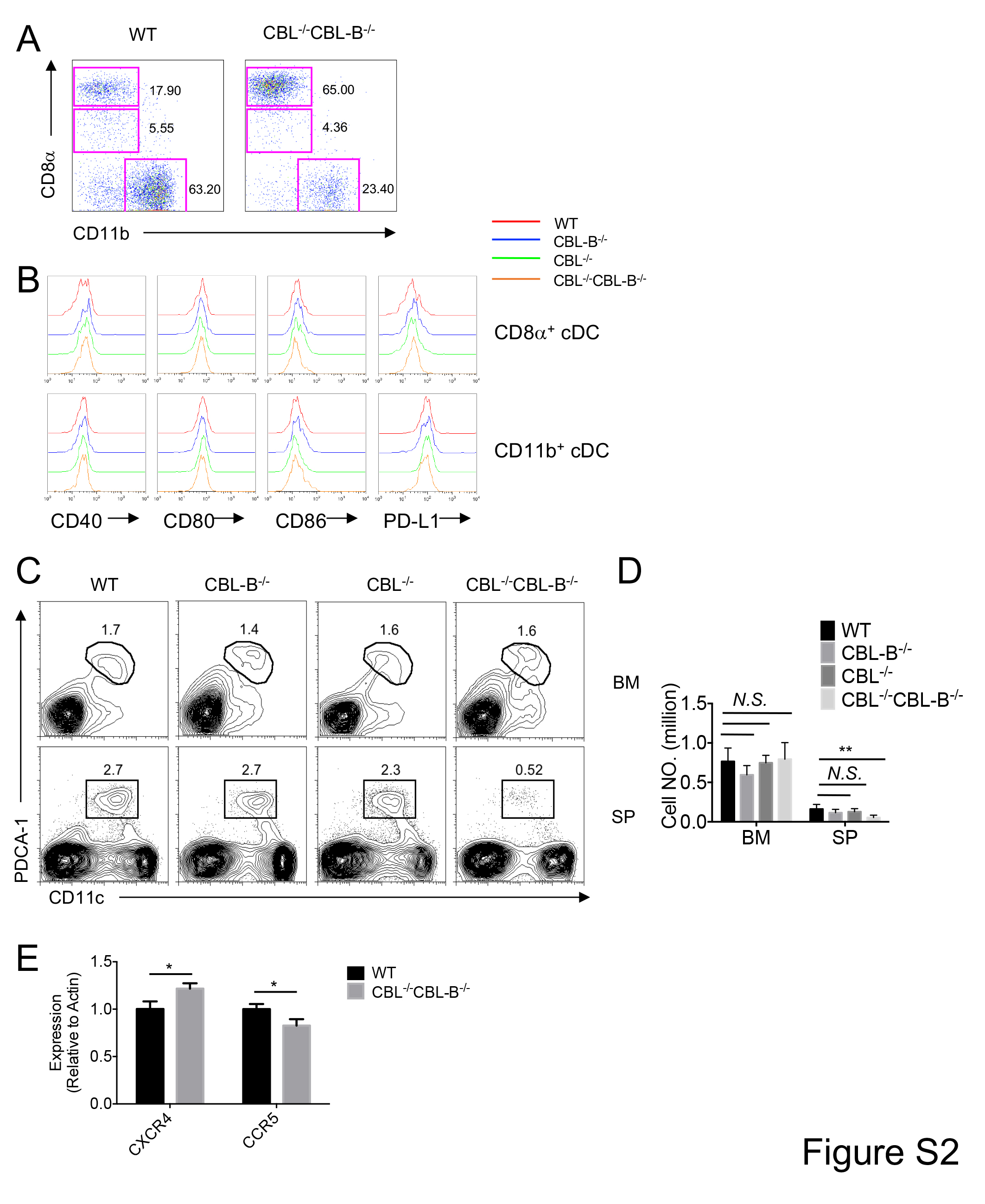

Supplement: Supplementary Figure 2 — Characterization of CD8a+ cDC1s and pDCs (A) CD8a vs CD11b staining of gated CD11c+ MHC-II+ splenic cDCs in WT and CBL-/-CBL-B-/- mice. The experiment was repeated for more than three times. (B) Expression of costimulatory ligands on CD8a+ cDC1s and CD11b+ cDC2s. The experiment was repeated for more than three times. (C, D) FACS analysis of the bone marrow (BM) and splenic (SP) pDCs. Shown are PDCA-1 vs CD11c staining of BM and spleen cells (C) and statistics (D). (n = 5). (E) qPCR analysis of CXCR4 and CCR5 expression in WT and CBL-/-CBL-B-/- pDCs. (n = 3). Data are means ± SEM of at least five mice or three independent experiments. *p < 0.01; **p < 0.001. [file Image_2.jpeg]

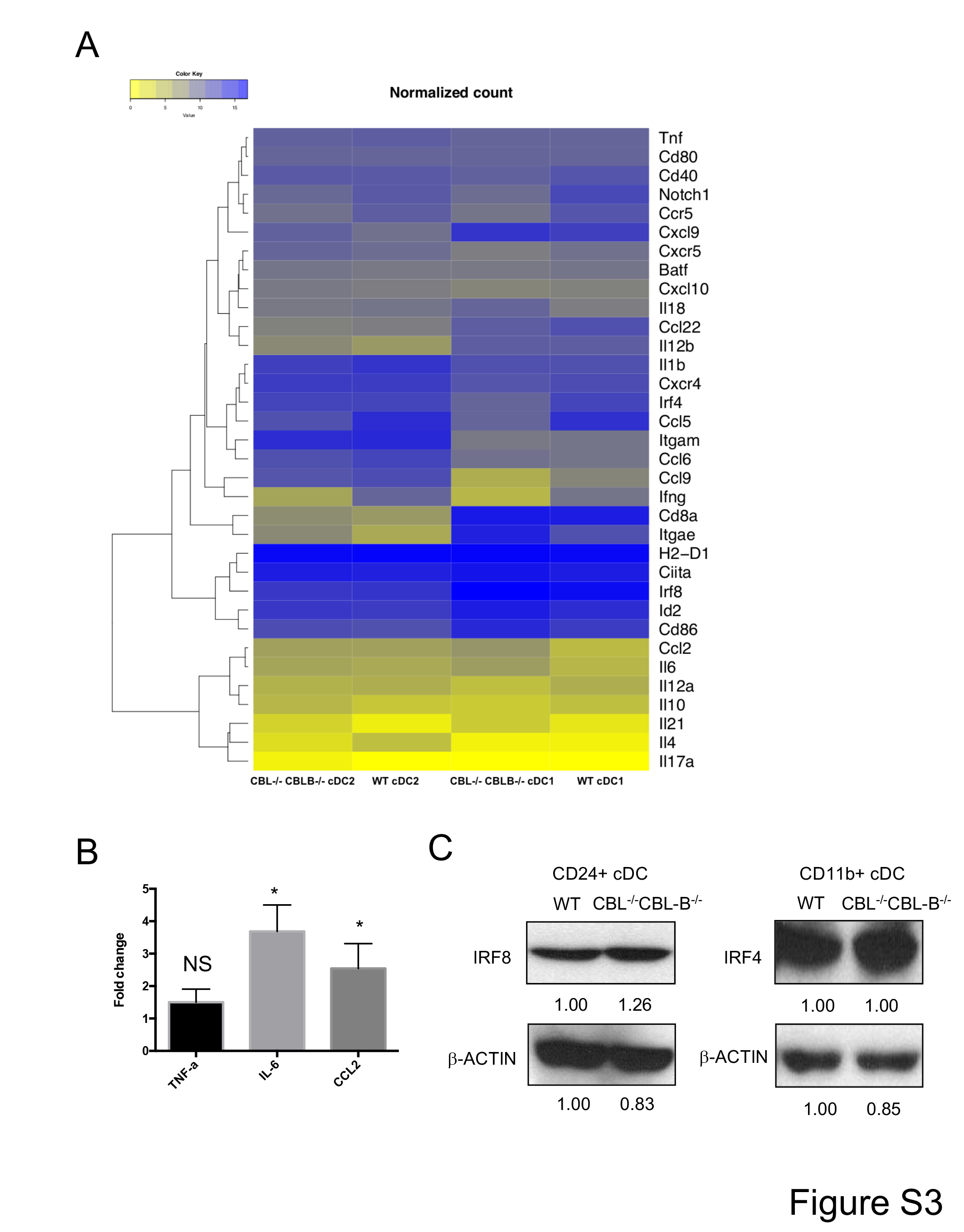

Supplement: Supplementary Figure 3 — Gene and protein expression profiles in WT and CBL-/-CBL-B-/- cDC1s and cDC2s (A) Heatmap analysis of the chemokines, cytokines and genes related to DC development and function. Data represent pooled RNA samples from 6x WT and 6x CBL-/-CBL-B-/- mice. (B) qPCR analysis of TNF-a, IL-6 and CCL2 expression in WT and CBL-/-CBL-B-/- CD8a+ cDC1s. Shown are fold increases of the corresponding gene transcripts in the mutant cells relative to WT cells. (n = 3). Data are means ± SEM of at least three independent experiments. *p < 0.01. (C) Western blot analyses of IRF4 and IRF8 in WT and CBL-/-CBL-B-/- CD24hi cDCs. [file Image_3.jpeg]

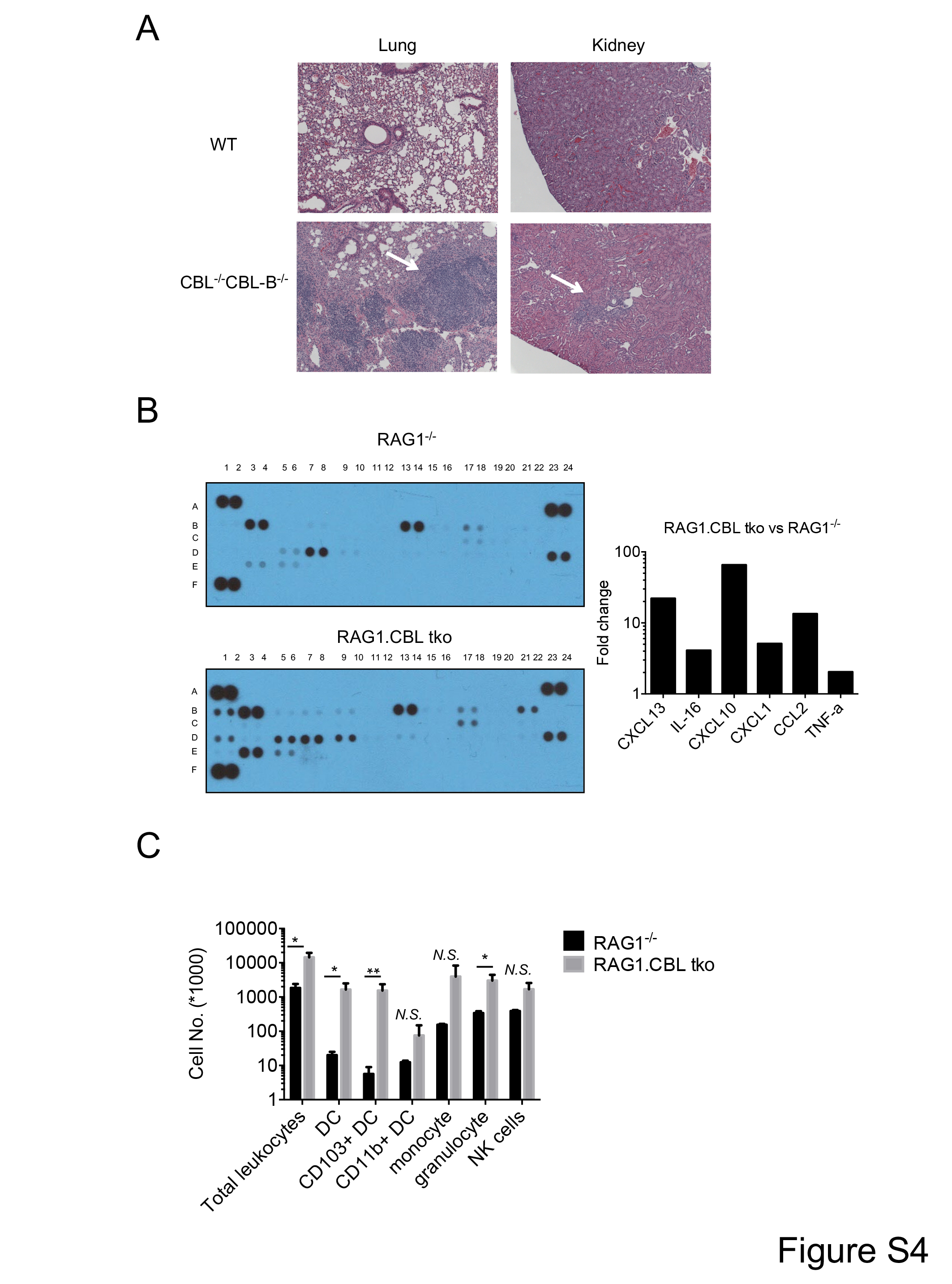

Supplement: Supplementary Figure 4 — Pathological analyses of CBL-/-CBL-B-/- mice and RAG1.CBL tko mice (A) H&E staining of lung and kidney sections of WT and sick CBL-/-CBL-B-/- mice. Data are from one of five mice. (B) Serum cytokine and chemokine titers. Shown are dot blot hybridization of serum cytokine and chemokine titers of pooled serum samples from WT RAG1-/- and sick RAG1.CBL tko mice. (n = 3). (C) Total numbers of inflammatory cell subsets in liver infiltrating leukocytes of RAG1-/- and sick RAG1.CBL tko mice. (n = 5). Data are means ± SEM of at least five mice. *p < 0.01; **p < 0.001. [file Image_4.jpeg]

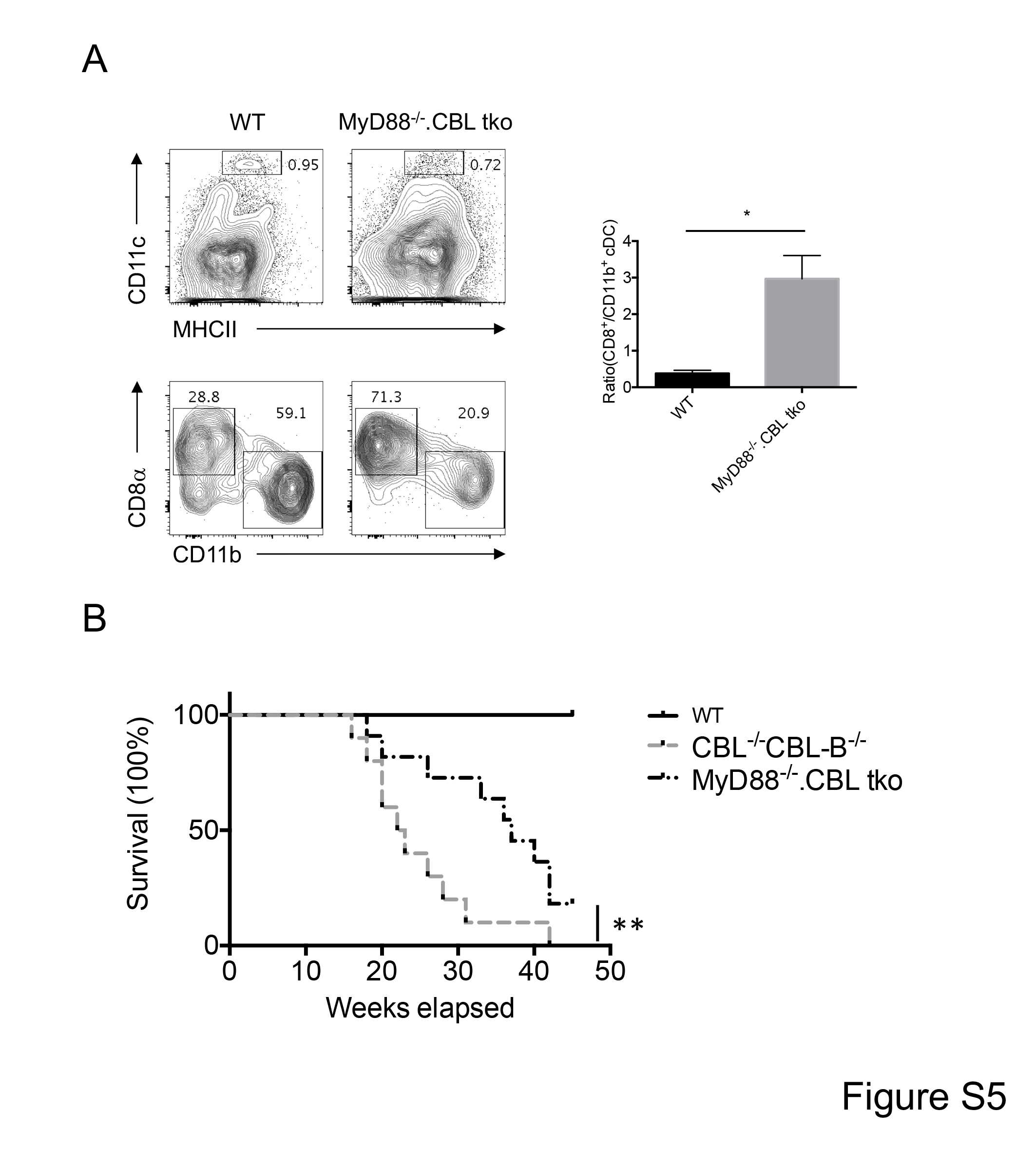

Supplement: Supplementary Figure 5 — Flow cytometric analysis of cDC subsets in and lifespan analysis of MyD88.CBL tko mice (A) FACS analysis (left) and statistics (right) of cDCs and CD8a+ cDC1s and CD11b+ cDC2s in WT (C57BL/6 or MyD88-/-) and MyD88.CBL tko mice. (n = 6). (B) Kaplan Meier survival analysis of WT (MyD88-/-), CBL-/-CBL-B-/- and MyD88.CBL tko mice. (n=10). Data are mean ± SEM of two independent experiments. *P < 0.01; **p < 0.001. [file Image_5.jpeg]
